# New bioresorbable wraps based on oxidized polyvinyl alcohol and leukocyte-fibrin-platelet membrane to support peripheral nerve neurorrhaphy: preclinical comparison *versus* NeuraWrap

**DOI:** 10.1038/s41598-019-53812-z

**Published:** 2019-11-20

**Authors:** Elena Stocco, Silvia Barbon, Veronica Macchi, Cesare Tiengo, Lucia Petrelli, Anna Rambaldo, Alessio Borean, Stefano Capelli, Andrea Filippi, Filippo Romanato, Pier Paolo Parnigotto, Claudio Grandi, Raffaele De Caro, Andrea Porzionato

**Affiliations:** 1 Institute of Human Anatomy, Department of Neurosciences, University of Padua, Via Gabelli 65, 35121 Padua, Italy; 2 LifeLab Program, Consorzio per la Ricerca Sanitaria (CORIS), Veneto Region, Via Giustiniani 2, 35128 Padova, Italy; 3 Clinic of Plastic Surgery, Department of Neurosciences, University of Padua, Via Giustiniani, 2, 35128 Padua, Italy; 4 Department of Immunohematology and Transfusion Medicine, San Martino Hospital, Viale Europa, 22, 32100 Belluno, Italy; 5 Department of Physics and Astronomy “G. Galilei”, University of Padua, Via Marzolo, 8, 35131 Padua, Italy; 6 LaNN, Laboratory for Nanofabrication of Nanodevices, Corso Stati Uniti, 4, 35127 Padua, Italy; 7 Foundation for Biology and Regenerative Medicine, Tissue Engineering and Signaling (TES) ONLUS, Via De Sanctis 10, Caselle di Selvazzano Dentro, 35030 Padua, Italy; 8 Department of Pharmaceutical and Pharmacological Sciences, University of Padua, Via Marzolo 5, 35131 Padua, Italy

**Keywords:** Peripheral nervous system, Regeneration and repair in the nervous system

## Abstract

Nerve wrapping improves neurorrhaphy outcomes in case of peripheral nerve injuries (PNIs). The aim of this preclinical study was to assess the efficacy of two novel biodegradable wraps made of a synthetic 1% oxidized polyvinyl alcohol (OxPVA) and a natural leukocyte-fibrin-platelet membrane (LFPm) *versus* the commercial product NeuraWrap. After rats sciatic nerve transection and neurorrhaphy, the wraps were implanted and compared for functional outcome, by sciatic function index assessment; structural characteristics, by histological/immunohistochemical analysis; ultrastructural features, by transmission electron microscopy. Moreover, a morphometric study was also performed and collagen distribution was observed by Second Harmonic Generation microscopy. After 12 weeks from implantation, all wraps assured nerve function recovery; no scar tissue/neuromas were visible at dissection. LFPm wraps were completely resorbed, while residues of OxPVA and NeuraWrap were observed. In all groups, biocompatibility was confirmed by the absence of significant inflammatory infiltrate. According to histological/immunohistochemical analysis and morphometric findings, OxPVA and LFPm wraps were both effective in preserving nerve integrity. These results assess that bioengineered OxPVA and LFPm wraps successfully guarantee favorable lesion recovery after PNI/neurorrhaphy and, in future, may be considered an interesting alternative to the commercial NeuraWrap.

## Introduction

To date, peripheral nerve injury (PNI) is considered a significant clinical challenge often resulting in impaired sensory and motor function^1^. This complex biological process and the related outcomes are strictly related to multiple factors; among these, axonal regeneration rate and misdirection extent, type of injury and injured nerve, level of the lesion, patient age and also compliance to training are included^2,3^. If sharp nerve transections occur, primary end-to-end nerve repair (i.e. neurorrhaphy) is the preferred surgical strategy for function restoring^4–7^ adjuvated by the environment of sensory and motor axons. However, even if surgery is perfectly adequate, to achieve a tensionless result is often disappointing and a complete functional recovery is difficult^8^. Intra-neural scarring with epineural thickening may occur, causing severe pain and dysfunction^9,10^. In fact, the cicatrix hampers the regeneration and disturbs the limb movement, as it acts as a mechanical barrier and forms adhesions with the surrounding tissues. Moreover, if left untreated, the scar may cause severe events; these include nerve oedema, ischemia and inflammatory changes up to nerve function loss.

These clinical features have prompted towards the development of supplementary procedures to perform after end-to-end suture, including wrapping of nerves^11–13^, with the aim to diminish inflammatory and immunologic reactions at the injury site^11,13,14^. Many different wraps have been considered in clinical practice; in addition to synthetic products, also collagen-based, fat, muscle, fascia have been used too^9,10^. In recent years, hemocomponents like platelet-rich-plasma (PRP) and platelet-rich-fibrin (PRF) have gained wide attention; however their effects on peripheral nerve regeneration are still controversial as reported in both preclinical and clinical studies^15–18^. Beside to applications of the PRP in the form of injectable gel^19,20^, in the literature its use as a fibrin membrane is also reported^21^.

In parallel, also resorbable synthetic wraps may be interestingly investigated. In a recent study, our attention focused on a novel biomaterial we developed that is 1% Oxdized Polyvinyl Alcohol (OxPVA)^22^, which demonstrated a promising *in vitro*^23^ and *in vivo*^24^ outcome when used as nerve-conduit. As discussed, the potential of the biomaterial may lie on the protein-loading ability of the polymer due to possible Schiff-base interaction between the amino-groups of neurotrophic substances released by the injured nerve and carbonyls of oxidized PVA. Moreover, it likely acts as a protectant towards the inflammatory molecules from the lesion-site and prevent the formation of adhesions discouraging cell adhesion.

In the light of this, the present study aimed to assess the efficacy of two innovative wraps, using an animal model of PNI. The wraps consisted of a leukocyte-fibrin-platelet membrane (LFPm)^25,26^ and OxPVA^22^. Their potential in promoting an optimal recovery of the lesion was compared with that of the collagen-based commercial product of bovine origin NeuraWrap (Integra LifeSciences, Plainsboro, NJ) (i.e. control group). NeuraWrap is indicated for the management of PNIs without substantial loss of nerve tissue; it acts as a nerve/surrounding interface, persisting in the implant site for up to 48 month. After surgery, the efficacy of the implanted wraps was evaluated in terms of functional recovery (at week 2 and 12), quality of nerve regeneration/repair, absence of inflammatory infiltrate and collagen deposition (at week 12). Verifying the behavior of the LFPm and OxPVA with respect to NeuraWrap may be also interesting as both LFPm and OxPVA could be encountered as potentially less expensive alternatives than the commercial counterpart.

## Results

### Neurorrhaphy and wraps implantation

**Figure 1:**
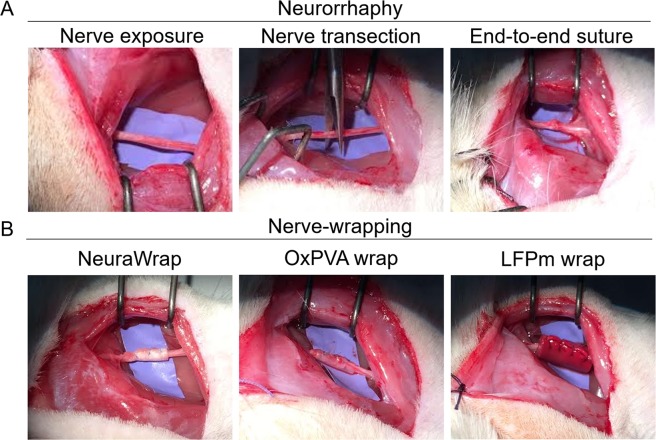
Animal model of peripheral nerve injury and wraps implantation. Neurorrhaphy (**A**) and nerve protectors implantation (**B**); in particular, NeuraWrap and wraps based on OxPVA and LFPm were used.

Surgical procedure of neurorrhaphy is showed in Fig. 1A. Thereafter, the animals were randomly implanted with NeuraWrap, OxPVA or LFPm (Fig. 1B) functioning like nerve protectors. Similarly to NeuraWrap, both OxPVA and LFP-based wraps also showed good handling properties during surgery. In fact, the materials were suturable and did not give rise to tears, thus ensuring their permanence at the implant site.

### Animal welfare after surgery

Surgery was well tolerated by rats of each experimental group. All animals showed a quick and adequate recovery after end-to-end neurorrhaphy and wraps implantation. Regular monitoring of animals conditions, including evaluation of normal activities, feeding patterns without weight loss, absence of wound infection or illness, proved their good health; moreover, autotomy did not occur along the study.

### Functional recovery

Sciatic Function Index (SFI) data showed a recovery in nerve function for all the implanted animals. At 2 weeks, the recorded values were 3.15 ± 0.52, 82.45 ± 1.55, 81.18 ± 2.25, 81.55 ± 2.15 for the non-operated foot, NeuraWrap group, OxPVA-wrap group and LFPm-wrap group, respectively. While, at 12 weeks, the recorded values were 2.25 ± 0.15, 52.14 ± 1.18, 46.5 ± 2.55, 49.46 ± 3.24 for the non-operated foot, NeuraWrap group, OxPVA-wrap group and LFPm-wrap group, respectively.

No significant differences were present between NeuraWrap group, OxPVA-wrap group and LFPm-wrap group at both end-points.

### Gross appearance of surgery site

Soon after euthanization and before samples excision, the surgery site was observed to preliminarily assess implants adequacy. Fascial and subcutaneous edema were not identified. Moreover, no signs of inflammation neither scar tissue or neuromas were visible.

**Figure 2:**
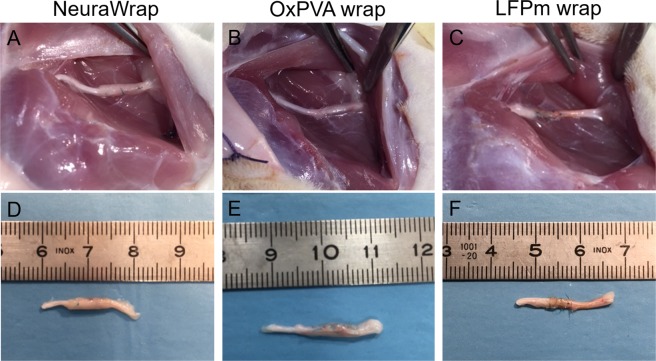
Gross appearance of surgical field and operated nerves explants at 12 weeks from surgery. *In situ* implant (**A–C**) and appearance after excision (**D–F**), of wraps based on NeuraWrap (**A**, **D**), OxPVA (**B**,**E**) and LFPm (**C**,**F**). After 12 weeks, no inflammation, scar tissue or neuromas were observed. Wrap residues were identified only for NeuraWrap and OxPVA while LFPm was resorbed.

Similarly to the NeuraWrap control group, sciatic nerves implanted with OxPVA showed the presence of wraps residues. Conversely, LFPm wraps were completely resorbed (Fig. 2).

### Characterization of the coaptation site

**Figure 3:**
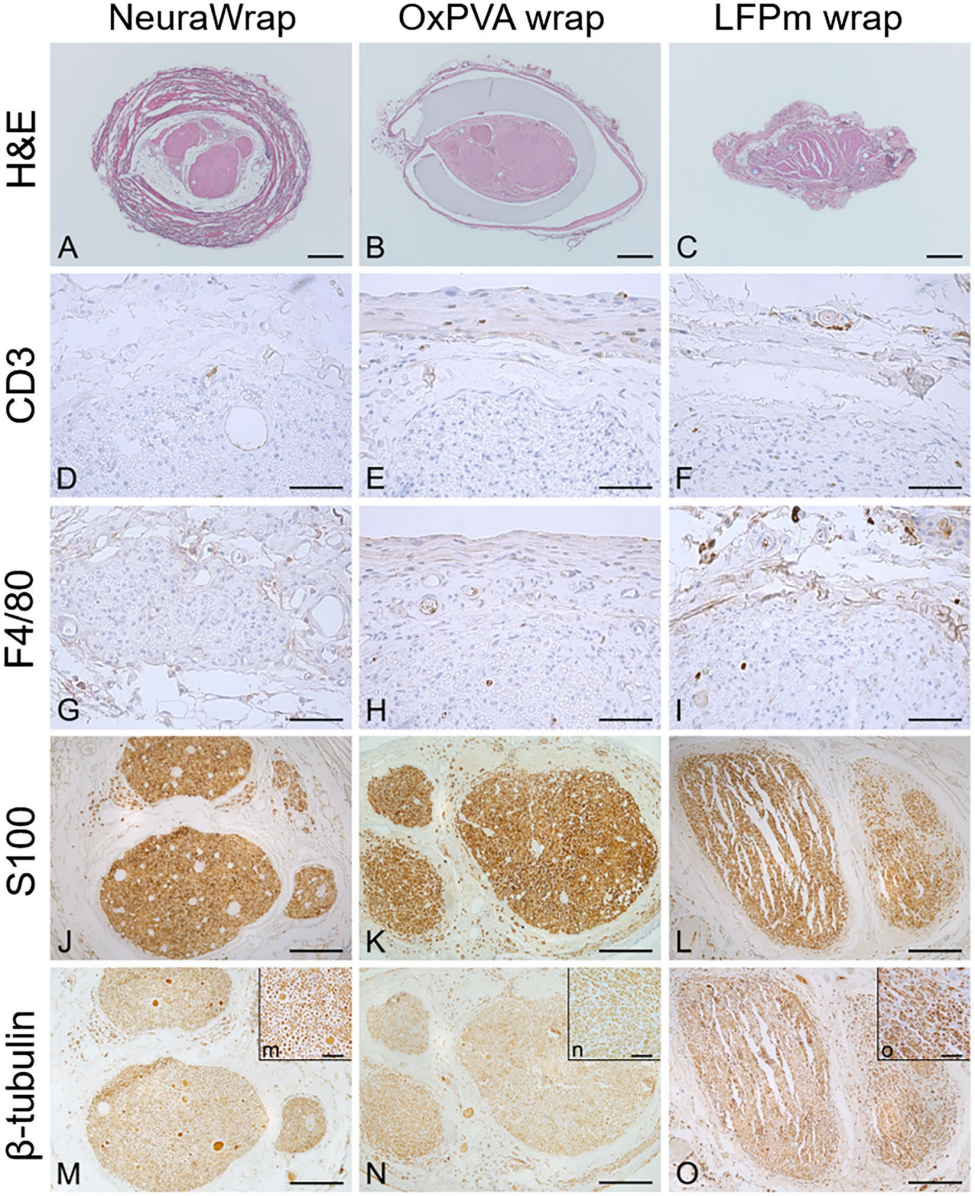
Histological and immunohistochemical analysis. Hematoxylin and eosin staining (H&E), anti-CD3, anti-F4/80, anti-S100 and anti-β-tubulin reactions performed on the coaptation site of Sprague-Dawley sciatic nerves implanted with NeuraWrap, OxPVA wraps and LFPm wraps. Analysis were occurred at 12 weeks from surgery (Scale bars: (**A–C**), 400 µm; (**D–I**), 50 µm; (**J–O**), 200 µm; scale bar in upper right insert (**m–o**) = 40 µm).

The suture-related holes, clearly identifiable in each H&E overall image, confirmed that the investigated cross-sections referred to the coaptation sites (Fig. 3).

For each experimental group, the H&E staining allowed to discriminate the morphology of the area of interest, also confirming previous evaluations according to gross-appearance.

NeuraWrap and OxPVA wraps were both identifiable after 12 weeks from surgery. In transversal section, NeuraWrap appeared like a thick layer with a fibrillar organization due to collagenous nature; adipose tissue was highlighted in the area between the nerve protector and the epineurium. OxPVA was like a transparent and dense layer surrounding the repaired nerve; structurally, the nerve showed the presence of a regular and homogeneous epineurium and regular fascicles organization. As observed *in situ* after dissection (Fig. 2), and in contrast to the other experimental groups, a thin fibrous capsule amenable to adherences was recognized around the OxPVA wraps. Regarding LFPm wraps, the nerve protector was completely resorbed and a thick and poorly organized epineurium was observed around the sectioned sciatic nerve. Differently from NeuraWrap group, adipose tissue was recognized in the space between the epineurium and the fascicles, which showed artefactual ruptures in the mid area. Moreover, according to H&E staining, no significant inflammatory infiltrate was visible in all experimental groups as also corroborated by anti-CD3 and anti-F4/80 immunohistochemical specific staining; in fact, only few CD3 and F4/80 positive cells were detected in all sample groups.

In addition, immunohistochemistry for S-100 and β-tubulin proved the ability of all nerve protectors in maintaining homogeneous distribution of the nerve fibers, without signs of degeneration after end-to-end neurorrhaphy (Fig. 3).

### Morphometric analysis

**Figure 4:**
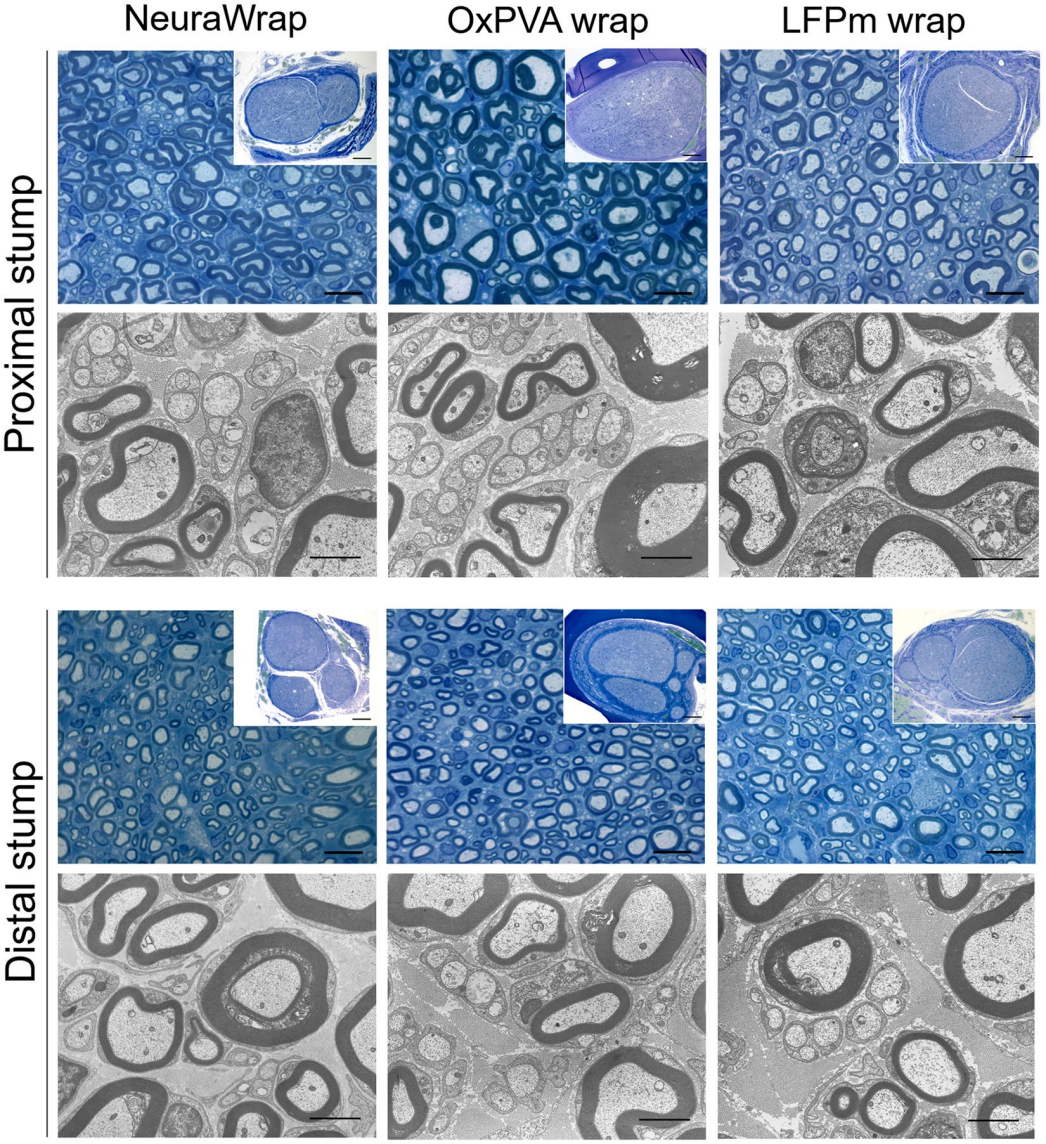
Characterization of Sprague-Dawley sciatic nerve explants at 12 weeks after surgery. Cross-sections of the proximal and distal portions of explants evaluated with Toluidine Blue staining (scale bars: 10 µm; scale bars in upper right insert: 200 µm) and Transmission Electron Microscopy (TEM) (scale bars: 2 µm).

Total cross-sections and fascicular areas, axons density and number of axons/fasci were assessed on semithin Toluidine Blue sections at both proximal and distal portion of the explants (Fig. 4).

**Figure 5:**
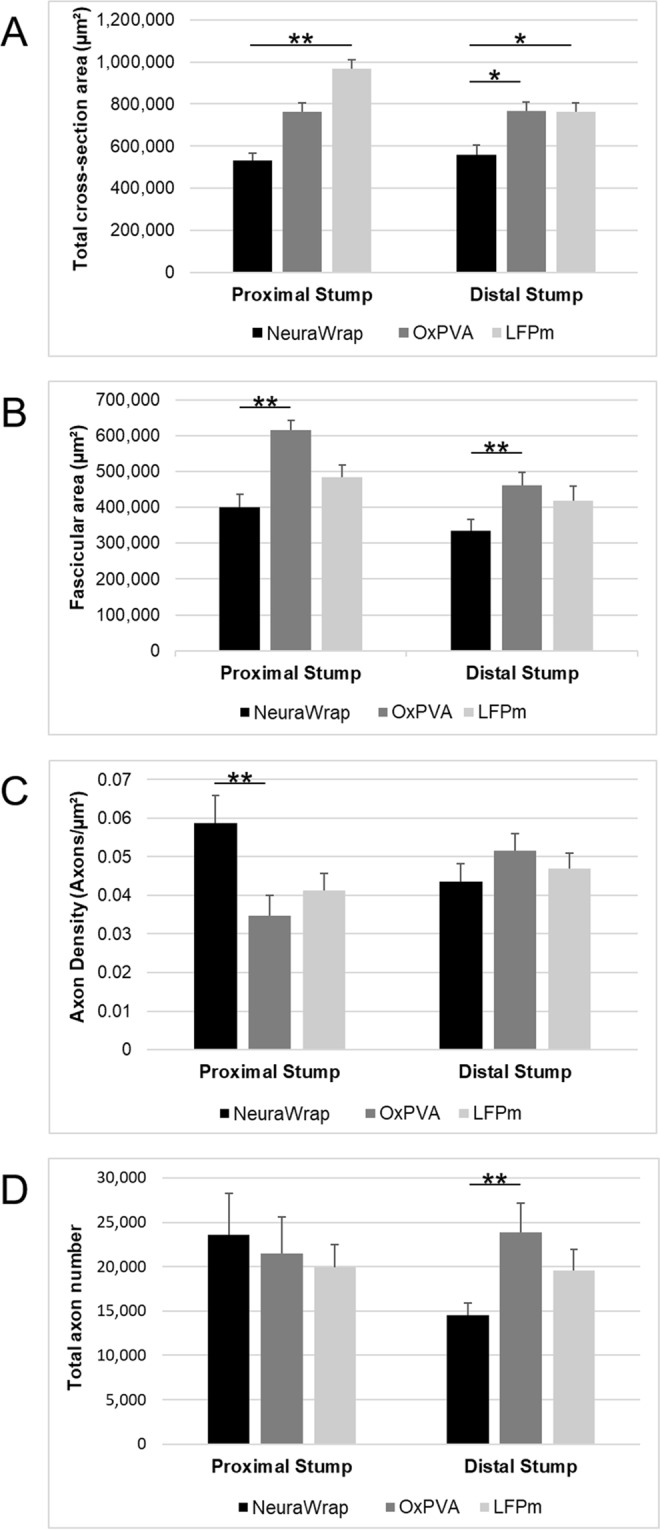
Morphometric assessment of repaired sciatic nerves at 12-weeks after surgery. Histograms show mean total cross-section nerve area (µm^2^), fascicular area (µm^2^), axon density (axons/ µm^2^) and total axons number of the proximal and distal portions of sciatic nerves implanted with NeuraWrap (control group) and wraps based on OxPVA and LFPm. Statistical analyses were performed by the Kruskal-Wallis test and Dunn’s multiple comparison test. Results are expressed as mean values ± SD (^*^p < 0.05; ^**^p < 0.01).

Morphometric data of the repaired sciatic nerves are represented in Fig. 5 being expressed as mean values ± standard deviation (SD).

The measurements of the total cross-section areas at the proximal stump showed significant difference between the LFPm-wrap group (967,318 ± 44,334 µm^2^) and NeuraWrap (533,176 ± 34,139 µm^2^; p < 0.01) but not compared to OxPVA (764,625 ± 42,364 µm^2^); while at distal level, both experimental groups had significantly higher (p < 0.05) values of mean total cross-section area (OxPVA wrap, 768,058 ± 41,555 µm^2^; and LFPm wrap, 762,634 ± 44,627 µm^2^) *versus* the commercial product NeuraWrap (558,021 ± 46,056 µm^2^).

Considering fascicular area (i.e. only fasci, without the connective tissue sheath around), OxPVA guaranteed significantly higher (p < 0.01) mean values compared to NeuraWrap at both proximal stump (615,080 ± 26,736 µm^2^ *versus* 400,061 ± 35,526 µm^2^) and distal level (461,577 ± 36,035 µm^2^ *versus* 334,427 ± 33,006 µm^2^); conversely, no significant differences were recorded for LFPm wraps at the two levels (proximal stump, 484,806 ± 34,325 µm^2^; distal stump, 418,437 ± 41,483 µm^2^).

Total axons density was significantly higher (p < 0.01) for NeuraWrap (0.059 ± 0.007/ µm^2^) *versus* OxPVA wraps (0.035 ± 0.005/µm^2^) but not *versus* LFPm wraps (0.041 ± 0.004/µm^2^) at proximal level; while, considering the distal portion, no differences among the groups aroused (NeuraWrap, 0.044 ± 0.005; OxPVA wrap, 0.052 ± 0.004; LFPm wrap, 0.047 ± 0.004).

Total number of axons was also determined and a significant difference was encountered only between OxPVA wraps (23,855 ± 3,314) and NeuraWrap (14,513 ± 1,416) at distal level (p < 0.01); conversely, no significant difference was observed with LFPm group (19,594 ± 2,386). At the proximal stump, there were 23,608 ± 4,628; 21,517 ± 4,106; 19,958 ± 2,487 mean total axons for NeuraWrap and the experimental groups OxPVA and LFPm, respectively; statistical analysis of the data revealed no significant difference between the groups.

Transmission Electron Microscopy (TEM) was used to qualitatively explore the ultrastructure of the explanted tissues at proximal and distal level. In all specimens, both myelinic and unmyelinic axons were recognized. No evident differences were highlighted between groups considering degenerated nerve fibers.

Representative Toluidine Blue staining image and TEM micrograph of the controlateral sciatic nerve are reported in Supplementary Material S1.

### Collagen distribution

**Figure 6:**
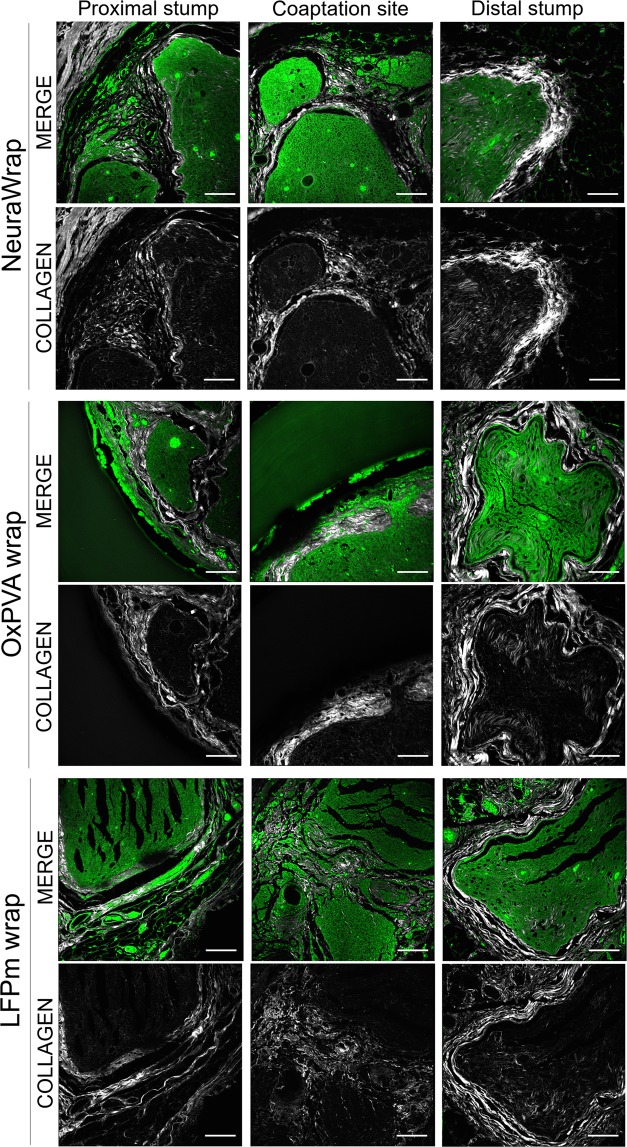
Second Harmonic Generation Microscopy of repaired sciatic nerves at 12 weeks after surgery. Cross-sections of the proximal portion, coaptation site and distal portion of NeuraWrap, OxPVA wraps and LFPm wraps explants, qualitatively evaluated by Second Harmonic Generation (SHG) microscopy (scale bars: 100 µm).

To detect structural and inflammatory response after neurorrhaphy and wraps implantation, collagen distribution at proximal stump, coaptation site and distal stump was observed by Second Harmonic Generation (SHG) microscopy (Fig. 6).

Considering the proximal stumps, the presence of a thin collagenous epineural tissue was confirmed in all the samples; for NeuraWrap and OxPVA wraps, collagen was also maintained among the fasci. Regarding the coaptation site, a thicker epineural tissue was observed for both NeuraWrap and OxPVA wraps; for LFPm wraps it appeared less organized and compact than the other specimens. Finally, all the distal stumps showed a well-recognizable epineural tissue which had a more filamentous appearance in OxPVA and LFPm wraps than in NeuraWrap.

Nervous tissue was preserved in all samples at each portion; however, the distal sections showed a collagenous infiltration slightly more intense.

Residues of the wraps were observed for all the specimens, particularly in correspondence of both the proximal stumps and the coaptation sites. In contrast to the synthetic OxPVA wraps, the collagenous fibril-like nature of NeuraWrap and LFPm wraps was evident.

Representative SHG images of the controlateral sciatic nerve are reported in Supplementary Material S1.

## Discussion

In this study, OxPVA and the hemocomponent LFPm were both tested as alternative biodegradable wraps in comparison to the commercial product NeuraWrap (Integra LifeSciencesCorp., www.integra-ls.com. Degradation: 36–48 months; diameter: 3–10 mm; lenght: 2–4 cm)^27^.

According to all gathered results, all materials showed adequate handling properties. They were easy to size according to the defect area suggesting their suitability for implantation in many different locations without “size fit” issues^28^.

Biodegradability eliminates the need for a second removal-surgery which is an important clinical aspect to consider for both the surgeon and the patient^28^. As described in previous characterization studies, both OxPVA and LFPm are biodegradable^22,24,25^ but differences were observed among the experimental groups. After 12 weeks from surgery, OxPVA wraps were still recognizable in the implant site. Despite appearing thinner and more transparent than the pre-implant sample, the flaps were still recognizable and paired without tears or translocation. This data is in accordance with previous findings referring to both OxPVA nerve conduits^24^ and disk-like scaffolds^22^, proving the occurrence of a non-complete 1% OxPVA biodegradation in 12 weeks. Conversely, LFPm wraps were completely resorbed, as previously shown by our group^25^ in the hemocomponent characterization study; no leavings of the membrane were visible at 21 day from subcutaneous implant.

Histological analysis by H&E allowed to compare the coaptation sites for their overall appearance; in contrast to OxPVA wrap, a certain coarctation was observed for both the LFPm wrap and NeuraWrap, to a lesser extent. Considering the different biodegradation profile of the wraps, correlation with the reabsorption rate may be realistic. As the synthetic OxPVA wrap remains longer in the defect area, it may mechanically protect the suture site from the surrounding tissues by preserving its integrity and structure. At 12 weeks, NeuraWrap continues to envelop the repair site, unlike LFPm wrap which was almost completely resorbed; Thus, these evidences may explain the different features described above between the groups.

Despite their distinct nature (i.e. synthetic and biological), all studied wraps assured for biocompatibility; in fact, only few CD3^+^ nor F4/80^+^ elements were recognized suggesting the absence of infiltrating lymphocytes and macrophages. Referring to LFPm wraps, these data are in accordance with the literature on matter. In fact, the LFPm are made of fibrin meshes, platelets and mesenchymal stem cell populations which are highly immuno-tolerated^29,30^ without causing rejection, necrosis or suppuration in the implantation site or in the surrounding tissues following implantation^31–35^. Controlling the inflammatory response at the suture-site influences injury-repair; it allows the nerve fibers for both faster crossing of neurorrhaphy site and regeneration of the early axons^36^. Supporting this assumption, for all experimental groups, the repaired tissues were positive to the specific markers for axons and Schwann cells (β-tubulin and S100, respectively) proving the preservation of nervous tissue^24,37^. Interestingly, the broad and homogeneous distribution of S100 positive elements proved no excessive fibroblastic response which would have compromised the proliferation of Schwann cells by creating a non-permissive environment^9,10^. These evidence were also furtherly supported by SHG microscopy images, morphometric analysis and functional recovery assessments.

Intriguing, SHG microscopy images validated and correlated with morphological characterization data^38^. For both OxPVA and LFPm derived samples, the epineural tissue and the inner part of each nerve section showed an appearance similar to that of NeuraWrap implanted nerves. All the specimens had characteristics compatible with that of a PNI undergoing to morpho-functional recovery with more consistent collagen at the epineural layer and traces of connective tissue inside the fascicular area. The absence of strong collagenous infiltration is an important finding as it may be considered an indicator of axonal damage severity and of how limited the capacity for successful axonal regrowth may be^38,39^. Likely, the intrinsic nature of the wraps supported the *in vivo* outcomes. Baltu *et al*.^40^ claimed that grafts containing collagen and non-adhesive surfaces (like NeuraWrap and OxPVA wraps, respectively) may be particularly advantageous for adequate axonal regeneration and repair also by limiting scar tissue formation and adhesions. Furthermore, Bastami *et al*.^15^ observed that hemocomponents enhance the microenvironment surrounding the lesion-site also increasing the number of regenerated nerve fibers.

Morphometric analysis, including regenerated nerve fibers counting, still remains the most frequently used method for outcome measure^28^. Wraps based on OxPVA guaranteed a significantly higher fascicular area *versus* NeuraWrap at both proximal and distal level. In contrast with NeuraWrap, this behavior may be ascribed to the mechanical protective role exerted by the synthetic polymer, limiting coarctation of the tissue which may obstacle adequate recovery after neurorrhaphy. In parallel, nerves implanted with OxPVA also showed a higher total axons number at the distal stump, despite similar axon density. These encouraging findings further validate the potential of OxPVA as a material for use in nerve recovery (i.e. manufacture of nerve conduits and wraps), implementing by positive outcome the preclinical evidences previously gathered^24^.

Regarding LFPm, the healing process showed nerves with a higher total cross-section area at both proximal and distal level than NeuraWrap; this thick epineurium could be mediated by the intrinsic nature of the hemocomponent. The fibrin fibers and growth factors support tissue healing especially soon after implantation; however, remaining in in the suture site for a limited period, they exert a reduced mechanical function than NeuraWrap. Despite that, comparable values of fascicular area, axon density and total axons number were observed at the two stumps *versus* the commercial product NeuraWrap, suggesting a similar behaviour and positive outcomes in preserving nerve fibers.

Similarly to NeuraWrap, the newly developed wraps based on OxPVA and LFPm successfully addressed the challenge of functional recovery assuring for a comparable outcome and SFI, along time. As reported by Fesli *et al*.^41^, the functional recovery after a nerve injury is referred not only to the regenerating axons number but also to a correct guidance towards their appropriate targets.

In the wide panorama of PNIs, this study mimics an ideal clinical situation as reparative surgery was performed soon after nerve transection. The surgical timing in nerve repair significantly affects the outcomes with better results when performed early^42,43^. However, there are clinical conditions where a delay in repair may occur, potentially resulting in poor function restoration. Thus, the suitability of OxPVA and LFPm wraps in late nerve repair could also be assessed in experimental animal models of suture at different time points (e.g. Jonsson *et al*.^44^). Moreover, also further structural, functional and morphometric evaluations on muscular recovery could contribute to a full comprehension of peripheral nerve regeneration and repair.

## Conclusions

The wraps based on OxPVA and LFPm both proved to sustain peripheral nerve regeneration/repair after sharp transection as showed by structural, ultrastructural and morphometric approaches. No inflammatory infiltration neither scar tissue formation was observed in correspondence or next to the implant site. Also functional analysis showed an outcome comparable to that of the NeuraWrap.

Thus, our results successfully demonstrated the existence of interesting alternatives to the expensive commercial products currently used in clinical practice. The high costs of collagen-based products like NeuraWrap are justified by the collagen-processing technologies (i.e. chemical and physical treatments for extraction and stabilization, use of additives and molding up to sterilization). In contrast, the production process to obtain OxPVA and LFPm is more simple and free from biological-risk issues. Moreover, as regards LFPm wraps, they can also be prepared extemporarily, according to the patient’s needs.

OxPVA wraps and LFPm wraps showed the desirable qualities of an envelope to be used in nerve repair. These are biodegradable materials that allow nerve gliding (without compressing the nerve), also creating an adequate environment that promotes axonal growth and limits intraneural scars at the repair site, which is often responsible of neuropathic pain^13^.

In particular, for OxPVA wraps a certain ability in contrasting coarctation at the suture site was also proved. Regarding LFPm wraps, experimental evidences were in accordance with the literature^28^ proving that the local application of exogenous agents with trophic properties properly boosts the regenerative capacity of injured nerves. In perspective, both OxPVA wraps and LFPm wraps may improve postoperative outcomes in case of sharp nerve transections. Moreover, the peculiar characteristics of these materials suggest the possibility for further studies focused on the development of bioactive wraps. Interestingly, OxPVA and LFPm could be combined together, obtaining a complex device which condenses the main features of both. In fact, consistent with our previous studies^22,23,45^, OxPVA may be functionalized/enriched with the hemo-derived stimulating factors, developing a smart platform actively involved in peripheral nerve healing.

## Methods

### Wraps set up

Three different wraps were compared: NeuraWrap; LFP based wraps and OxPVA-wraps. The commercial product NeuraWrap was purchased by Integra; as concerns LFP and OxPVA wraps they were developed according to standardized protocols. In particular, OxPVA membranes were manufactured from an oxidized PVA polymeric solution which was obtained through a controlled chemical oxidative reaction^22^. The OxPVA solution was then poured into the mould (i.e. two sheets of glass, separated by a 3 mm thick steel spacer) and crosslinked by freezing (at −20 °C for 24 h)/thawing (at 2.5 °C for 24 h) (FT); seven FT cycles occurred to obtain the membranes which were then stored at −20 °C until use. Prior to implant, the OxPVA wraps were disinfected (70% alcohol) and carefully washed with phosphate buffered saline (PBS 0.1 M, pH 7.4).

The LFP wraps were set up from LFP membranes developed according to Barbon *et al*.^25^ briefly, two basic hemocomponents (CLP and plasma) were collected and after plasma cryoprecipitaton they were mixed, setting platelets within a range of 1 000–2 000 × 10^3^/µl. The final mixed hemocomponent (leukocyte-platelet concentrate mixed with cryoprecipitate, CLP-M) was activated by calcium gluconate and centrifuged to achieve membrane formation.

### Surgical procedure

All animal procedures were approved by the ethical committee of Padua University (D.M. n.162/2013-B), according to the Italian Department of Health guidelines.

Thirty Sprague-Dawley rats were randomly divided into three experimental groups (n = 10/each): (a) NeuraWrap; (b) OxPVA; (c) LFPm. A gas mixture of isoflurane/oxygen was used to induce anesthesia; then, after preparing the surgical field, the sciatic nerve was exposed according to a gluteal-splitting approach and transected with microsurgical scissors. Nylon 8–0 sutures were performed to connect the epineuria of the proximal and distal stumps and the nerve protectors were trimmed and wrapped around the site of repair. Each wrap was 1 cm in length and 1 mm in thickness; moreover, the nerve protectants were secured along their length by interrupted Nylon 8–0 sutures as well. The incision was closed in layers using 4–0 silk sutures. After surgery, adequate anti-inflammatory (Rimadil, 5 mg/kg) and antibiotic (Bytril, 5 mg/kg) therapies were administered for 5 days and the rats recovered in the cage being housed in a temperature-controlled facility and fed with laboratory rodent diet and water *ad libitum*.

The final end-point was set-up at 12 weeks after surgery; euthanasia by carbon dioxide asphyxiation was performed. Hence, the implants were excised and adequately fixed for histological/immunohistochemical (n = 5 samples/group) and TEM (n = 5 samples/group) analysis.

### Sciatic Functional Index

At 2 and 12 weeks after surgery, the animals underwent to a sciatic function test aiming to assess the functional status of the operated sciatic nerve. A gangway (100 cm long/10 cm wide) lined with white paper was set-up; rats with their feet stained with black ink were placed at the beginning of it and allowed to walk up thereby. For each rat, five measurable footprints were considered for the calculation of SFI according to the formula by Bain *et al*.^46^.

$$SFI=-\,38.3\frac{EPL-NPL}{NPL}+109.5\frac{ETS-NTS}{NTS}+13.3\frac{EIT-NIT}{NIT}-8.8$$

Print length (PL) corresponds to the distance from the heel to the top of the third toe, toe spread (TS) stands for the distance from the first to the fifth toe and the intermediary toe spread and (IT) represents the distance between the second and the fourth toe. NPL, NTS and NIT represent the PL, TS and IT recorded from the non-operated foot, respectively. EPL, ETS, and EIT represent the PL, TS and IT recorded from the operated foot (E, experimental side), respectively.

### Macroscopic evaluation

At 12 weeks from surgery, dissection occurred and a surgeon performed a blind evaluation of the macroscopic aspect of the neurolysis sites. In particular, the analyzed aspects comprehended the quality of wound healing, the presence of inflammation signs and perineural adhesion.

### Histological and immunohistochemical characterization

**Figure 7:**
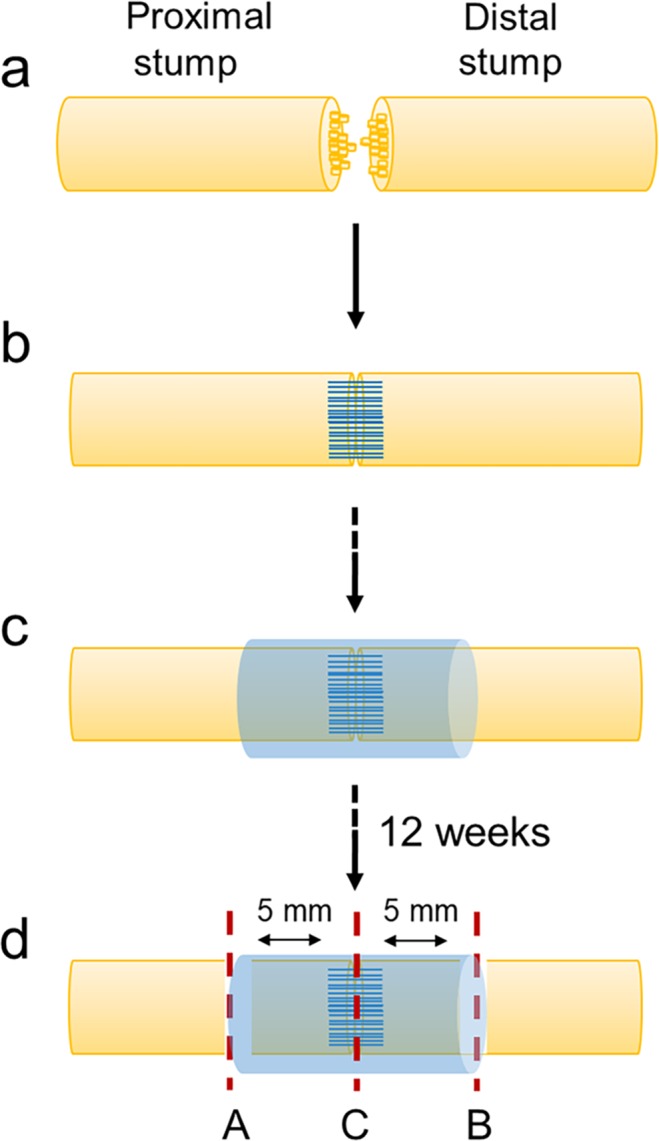
Experimental design and explants sampling for histological and immunohistochemical characterization and morphometric analysis by Transmission Electron Microscope. The image represents the surgical procedure, including sharp transection (**a**), coaptation by end-to-end neurorrhaphy (**b**) and wrap implantation (**c**). After 12 weeks, sciatic nerves sectioning occurred before histological and immunohistochemical analysis, Transmission Electron Microscopy and Second Harmonic Generation (SHG) studies. In (**d**), the investigated areas are showed in correspondence to the red-dotted lines. The coaptation site (C) was analyzed by both histology (haematoxylin and eosin) and immunohistochemistry (anti-CD3, anti-F4/80, anti-S100, anti-β-tubulin). The portions next to the coaptation site (**A** and **B**, from the nearest area to the most distant area, respectively) were analyzed by Toluidine-Blue staining and TEM. All portions were visualized by SHG.

The explanted samples corresponding to the repaired nerves were fixed in 10% formalin in PBS soon after removal. Hence, they were cross-cut in the middle portion (coaptation site) and retrogradely cut into 4 μm-thick serial sections after paraffin embedding. Hematoxylin and eosin (HE) staining occurred according to routine protocols. In parallel, immunological characterization was performed with the following antibodies diluted in PBS: anti-CD3 (polyclonal rabbit anti-human CD3, A 0452; Dako, Milan, Italy) diluted 1:500; anti-F4/80 (polyclonal rabbit anti-mouse anti-F4/80, sc-26643-R; Santa Cruz Biotechnology, CA, USA) diluted 1:800; anti S-100 (polyclonal rabbit anti-S100, Z 0311; Dako) diluted 1:5000; anti-β-tubulin (polyclonal rabbit neuronal class III β-tubulin, PRB-435P; Covance, Princeton, NJ, USA). Except for S-100, antigen unmasking was performed with 10 mM sodium citrate buffer, pH 6.0, at 90 °C for 10 min. The sections were then incubated for 30 min in blocking serum [0.04% bovine serum albumin (BSA; A2153, Sigma-Aldrich, Milan, Italy) and 0.5% normal goat serum (X0907, Dako)] to eliminate unspecific binding, and then incubated for 1 h at RT with the above primary antibodies. Primary antibody binding was revealed by incubation with anti-rabbit/mouse serum diluted 1:100 in blocking serum for 30 min at RT (Dako® EnVision + TM peroxidase, rabbit/mouse; Dako, Glostrup, Denmark) and developed in 3,3′-diaminobenzidine for 3 min at RT. Lastly, the sections were counterstained with haematoxylin. As a negative control, sections were incubated without primary antibodies (Fig. 7).

### Ultrastructural analysis

To evaluate the contribution of the wraps in tissue regeneration effectiveness, ultrastructural features (i.e. axons distribution and tissue quality) of the operated sciatic nerves were analyzed at 12 weeks from surgery. Explanted samples were fixed in 2.5% glutaraldehyde in 0.1 M PBS; thereafter, each nerve was sectioned eight millimetres distal and proximal from the coaptation site. Sections, post-fixed in 1% osmium tetroxide (Agar Scientific Elektron Technology - UK) in 0.1 M phosphate buffer, were dehydrated in a graded alcohol series and embedded in Epoxy resin according to routine protocols. Semi-thin sections (0.5 µm) were cut with an ultramicrotome RMC-PTX PowerTome **(**Boeckeler Instruments, Arizona-USA) before staining with with 1% Toluidine Blue. A Leica DMR microscope (Leica Microsystems Wetzlar- Germany) was used to acquire images; further images analysis were performed using ImageJ image processing software (National Institutes of Health, Bethesda, MD) for blinded analysis.

Ultrathin sections, 60 nm, were collected on 300-mesh copper grids, counterstained with 2% uranyl acetate and then with Sato’s lead. Specimens were observed by a Hitachi H-300 TEM (Fig. 7).

### Morphometric study

Morphometric analysis was performed on semithin Toluidine Blue-stained sections belonging to the proximal and distal portions of each sample, as previously described^24^. Two areas of the proximal and distal stumps, were analyzed, as described in Fig. 7. Briefly, after measuring total cross-section area and fascicular area of each portion, 5 quadrants in the fascicular area were randomly identified; then, 3 high-power fields (100x) of equal area from each quadrant were analyzed for myelinated and unmyelinated axons. Average axon density was determined dividing total axon number by the area sampled.

### Collagen deposition

Neural collagen deposition after 12 weeks from surgery was imaged using label-free microscopy for a qualitative comparison with data from histology, immunohistochemistry and TEM.

Specimens were processed as previously described for histology and immunohistochemistry. Briefly, after fixation in 10% formalin in PBS and paraffin embedding, 4 μm-thick serial sections were cut and deparaffinized according to routine protocols. SHG imaging was performed using a custom developed multiphoton microscope, described in detail elsewhere^47^. An incident wavelength of 800 nm (~40 mW average laser power measured under the microscope objective) was used in order to detect the collagen’s SHG signal at 400 nm, while simultaneously excite the two-photon autofluorescence signal from the elastin and the intrinsic fluorophores within the nerves (collected by a PMT inside the operative wavelength range of 505–545 nm). The images were acquired at a fixed resolution of 1024 × 1024 pixels, averaged over 120 consecutive frames, with a pixel dwell time of 0.14 μs.

### Statistical analysis

Statistical analyses were performed by the Kruskal-Wallis test and Dunn’s multiple comparison test. Results were expressed as mean ± SD. *P* < 0.05 was considered to be statistically significant. Statistical calculations were carried out by Prism 3.0.3 (GraphPad Software, San Diego, CA).

## Supplementary information

Dataset 1
