# Supplementary material for: New bioresorbable wraps based on oxidized polyvinyl alcohol and leukocyte-fibrin-platelet membrane to support peripheral nerve neurorrhaphy: preclinical comparison versus NeuraWrap
Source: Sci Rep. 2019 Nov 20;9:17193. doi: 10.1038/s41598-019-53812-z (PMC6868173; doi:10.1038/s41598-019-53812-z)
Supplement: Supplementary file 1 — Dataset 1 [file 41598_2019_53812_MOESM1_ESM.pdf]

New bioresorbable wraps based on oxidized polyvinyl alcohol and leukocyte-fibrin-platelet membrane to support peripheral nerve neurorrhaphy: preclinical comparison *versus* NeuraWrap

Elena Stocco, Silvia Barbon, Veronica Macchi, Cesare Tiengo, Lucia Petrelli, Anna Rambaldo, Alessio Borean, Stefano Capelli, Andrea Filippi, Filippo Romanato, Pier Paolo Parnigotto, Claudio Grandi, Raffaele De Caro and Andrea Porzionato

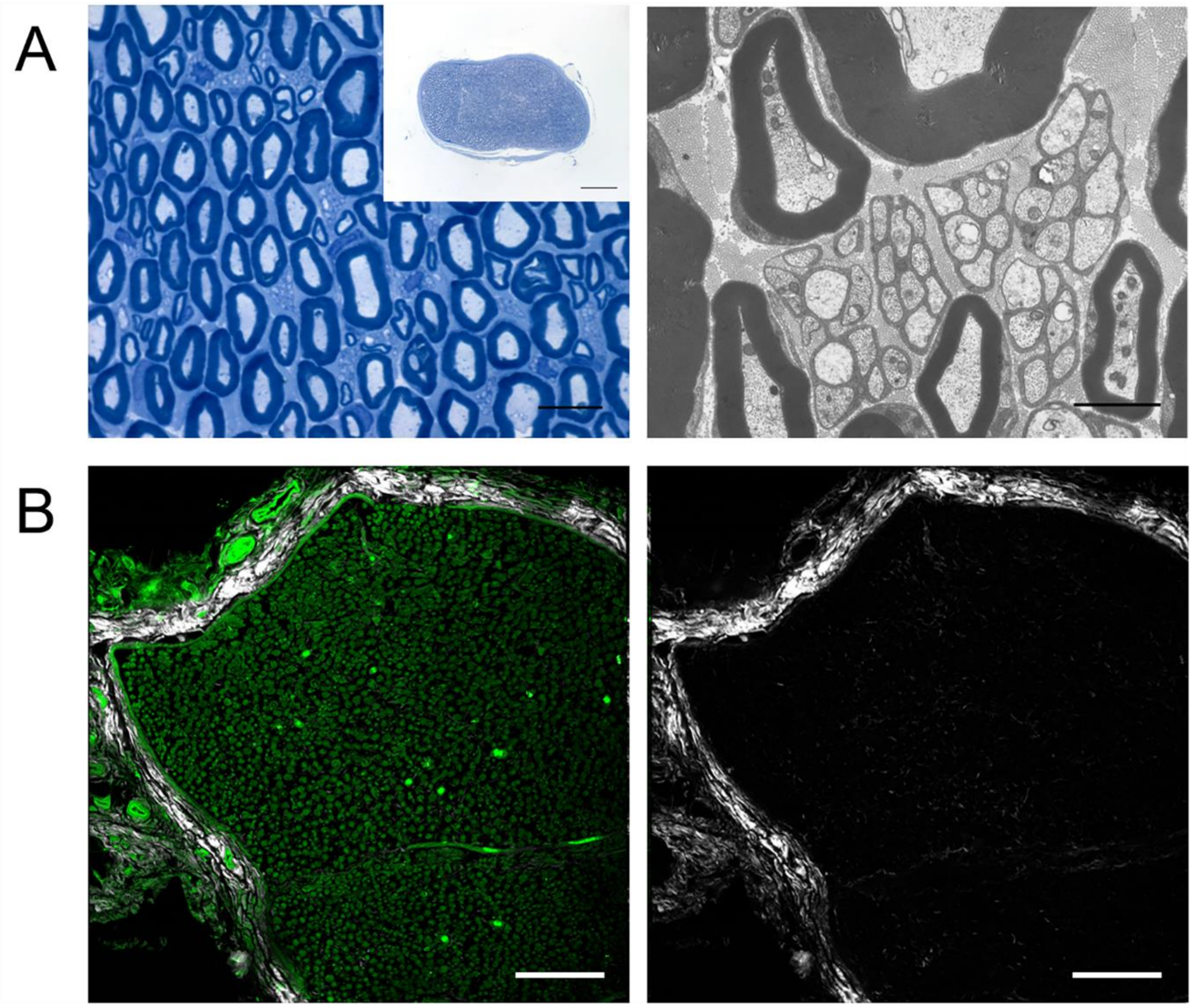

Supplementary figure S1. Structure of a non-operated controlateral rat sciatic nerve. (A) Representative cross-sections of the controlateral sciatic nerve evaluated with Toluidine Blue staining (scale bars: 10 µm; scale bar in upper right insert: 200 µm), Transmission Electron Microscopy (TEM) (scale bars: 2 µm) and (B) Second Harmonic Generation (SHG) microscopy (scale bars: 100 µm).
